# Causal relationship between gut microbiota and adenomyosis: metagenomics sequencing and Mendelian randomization

**DOI:** 10.3389/fcimb.2026.1772864

**Published:** 2026-06-08

**Authors:** Chunxi Tang, Bojuan Li, Jian Chen, Xiaoyun Liu, Chaokun She

**Affiliations:** 1 Department of Gynecology, Shenzhen Baoan Women’s and Children’s Hospital, Shenzhen, China; 2 Department of Gynecology, Guizhou Maotai Hospital, Zunyi, China; 3 Zunyi Medical University and Science and Technology College, Zunyi, China; 4 Department of Laboratory Medicine, The First People’s Hospital of Zunyi, The Third Affiliated Hospital of Zunyi Medical University, Zunyi, China

**Keywords:** adenomyosis, gut microbiota, high-throughput sequencing, Mendelian randomization, metagenomics

## Abstract

**Background:**

Emerging evidence implicates the gut microbiota in the pathogenesis of adenomyosis (AM); however, whether this association is causal and through which mechanisms it operates remain largely unknown.

**Methods:**

To interrogate potential causal relationships, we performed a two-sample Mendelian randomization (MR) analysis leveraging inverse-variance weighting (IVW) as the primary estimator, complemented by MR-Egger, weighted median, and weighted mode approaches, to evaluate the causal effects of gut microbial taxa and microbiota-derived metabolic pathways on AM. We further conducted mediation analyzes to delineate the role of circulating immune-cell phenotypes in this process. In parallel, in an independent clinical cohort, 22 patients with AM and 23 age-matched healthy controls recruited from the health-screening center of our institution were enrolled according to stringent inclusion and exclusion criteria (including antibiotic-use history and long-term local residency) and subjected to shotgun metagenomic sequencing. Significant differences in the types of bacterial communities were observed between the AM group and the control group. Subsequently, the results were cross-compared with those of the MR study using the Linear Discriminant Analysis Effect Size (LEfSe) method, and further verified using the ANCOM-BC method to determine the common microbial characteristics.

**Results:**

MR analysis identified ten microbial taxa and ten metabolic pathways with evidence of potential causal associations with AM. Of these, nine taxa and five pathways were associated with a reduced risk of AM, including *Alistipes indistinctus* (OR = 0.847, 95% CI = 0.754–0.951, *p* = 0.005, *p*~FDR~ > 0.05), *Ruminococcus torques* (OR = 0.818, 95% CI = 0.712–0.941, *p* = 0.005, *p*~FDR~ > 0.05), class *Deltaproteobacteria* (OR = 0.780, 95% CI = 0.629–0.967, *p* = 0.024, *p*~FDR~ > 0.05), family *Desulfovibrionaceae* (OR = 0.780, 95% CI = 0.629–0.967, *p* = 0.024, *p*~FDR~ > 0.05), order *Desulfovibrionales* (OR = 0.780, 95% CI = 0.629–0.967, *p* = 0.024, *p*~FDR~ > 0.05), *Parasutterella excrementihominis* (OR = 0.875, 95% CI = 0.784–0.977, *p* = 0.017, *p*~FDR~ > 0.05), *Ruminococcus bromii* (OR = 0.836, 95% CI = 0.718–0.972, *p* = 0.020, *p*~FDR~ > 0.05), *Bacteroides finegoldii* (OR = 0.919, 95% CI = 0.855–0.987, *p* = 0.020, *p*~FDR~ > 0.05), and the genus *Parasutterella* (OR = 0.886, 95% CI = 0.797–0.986, *p* = 0.026, *p*~FDR~ > 0.05); the five protective pathways comprised dTDP-L-rhamnose biosynthesis (OR = 0.819, 95% CI = 0.674–0.995, *p* = 0.045, *p*~FDR~ > 0.05), lactose and galactose degradation (OR = 0.818, 95% CI = 0.689–0.972, *p* = 0.022, *p*~FDR~ > 0.05), the reductive TCA cycle (OR = 0.919, 95% CI = 0.851–0.993, *p* = 0.032, *p*~FDR~ > 0.05), allantoin degradation to glyoxylate (OR = 0.907, 95% CI = 0.830–0.991, *p* = 0.030, *p*~FDR~ > 0.05), and glycolysis I (from glucose-6-phosphate) (OR = 0.850, 95% CI = 0.747–0.967, *p* = 0.013, *p*~FDR~ > 0.05).

Conversely, one taxon and five pathways were associated with an increased risk of AM: the genus *Lactobacillus* (OR = 1.083, 95% CI = 1.008–1.164, *p* = 0.030, *p*~FDR~ > 0.05), degradation of glucose and glucose-1-phosphate (OR = 1.202, 95% CI = 1.056–1.369, *p* = 0.005, *p*~FDR~ > 0.05), peptidoglycan biosynthesis (in Enterococcus faecium) (OR = 1.138, 95% CI = 1.007–1.285, *p* = 0.039, *p*~FDR~ > 0.05), pyruvate fermentation to acetone (OR = 1.118, 95% CI = 1.001–1.248, *p* = 0.048, *p*~FDR~ > 0.05), glycerol degradation to butanol (OR = 1.118, 95% CI = 1.011–1.237, *p* = 0.031, *p*~FDR~ > 0.05), and *de novo* pyrimidine deoxyribonucleotide biosynthesis (OR = 1.216, 95% CI = 1.063–1.390, *p* = 0.004, *p*~FDR~ > 0.05).Mediation analysis revealed that the immune phenotype “CD24 on CD24^+^CD27^+^ B cells” mediated the pathway from *Ruminococcus bromii* to AM, accounting for 32.91% of the total effect (*p* = 0.020).Shotgun metagenomic profiling of the clinical cohort demonstrated no significant differences in α-diversity or β-diversity between the AM and control groups. At the phylum level, the relative abundance of Desulfobacterota was significantly decreased in the AM group (*p*< 0.05), and at the genus level, *Alistipes* was similarly reduced (*p*< 0.05). LEfSe analysis further indicated enrichment of *Escherichia* and *Clostridium* in the AM group, whereas *Desulfobacterota* and *Rikenellaceae* were enriched in the Control group. Matching the aforementioned results with the Mendelian randomization (MR) outcomes revealed that *Desulfovibrionales* and *Desulfovibrionaceae* constituted the shared microbial taxa. This finding was subsequently re-validated and confirmed using the ANCOM-BC method.

**Conclusions:**

Integrating genetic causal inference with clinical metagenomic validation, this study provides convergent evidence that specific gut microbial taxa, their associated metabolic pathways, and immune-cell–mediated mechanisms may be causally implicated in the development of AM. These findings offer a framework for future microbiota-targeted preventive and therapeutic strategies against AM.

## Introduction

Adenomyosis(AM) is a common gynecological disorder characterized by the presence of endometrial glands and stroma within the myometrium, and it commonly presents with abnormal uterine bleeding, pelvic pain, and infertility (Upson and Missmer, 2020). The reported prevalence of AM among women undergoing hysterectomy ranges from 20% to 34%, largely reflecting heterogeneity in diagnostic criteria and interobserver variability among pathologists (Kho et al., 2021). Although the etiology and pathophysiology remain incompletely understood, several mechanisms have been proposed. One central mechanism involves endometrial invasion at the endometrial-myometrial junction. This invasion is thought to initiate tissue injury-repair responses, amplify local inflammation, and increase intralesional estrogen levels. Under myometrial hypoxia, platelet aggregation activates HIF-1α. This pathway facilitates ectopic endometrial gland formation and sustains stromal hypoxia. In parallel, activation of NF-κB and/or TGF-β1 upregulates StAR, HSD3B2, aromatase, and HSD17B1 in ectopic endometrial stromal cells. This steroidogenic shift increases local estrogen production and promotes epithelial-mesenchymal transition in endometrial epithelial cells (Guo, 2022, 2023).

Alternative mechanisms proposed for the development of AM include metaplasia of endometrial stem cells within the myometrium and metaplasia arising from Müllerian duct remnants (Guo, 2022, 2023). Recent genomic studies indicate that KRAS-mutated ectopic endometrial cell clones exhibit increased invasiveness and proliferation. These traits facilitate myometrial infiltration and contribute to progesterone resistance (Bulun et al., 2023). Furthermore, dysregulated immune responses have been implicated; one study found decreased levels of Th17 and Treg cells in women with AM compared to healthy controls (Bourdon et al., 2021). The pathogenesis of AM seems to entail a multifaceted interaction of hormonal imbalances, genetic predispositions, and immune dysregulation (Moawad et al., 2023). Hysterectomy remains the most common surgical intervention. Laparoscopic and open approaches are commonly employed as procedural alternatives. In the course of pharmacotherapy treatments, more than 72% of patients use analgesics, whereas a smaller proportion receive hormonal therapy. These patterns impose substantial burdens on patients and healthcare systems. Effective long-term pharmacotherapy options remain limited (Yu et al., 2020). Therefore, the identification of risk factors and the development of innovative preventive and therapeutic approaches are crucial.

With the progress in sequencing technologies, the concepts of the gut-uterus axis and gut-vagina axis have emerged and garnered increasing attention. Current evidence suggests that estrogen aggregates are closely associated with the gut-vagina axis and may also be linked to the gut-uterus axis (Takada et al., 2023). Because endometrial hyperplasia and endometrial cancer depend on estrogen signaling, these estrogen aggregates may regulate disease progression via the aforementioned axes and thereby influence the occurrence and progression of estrogen-dependent gynecologic disorders (Takada et al., 2023). As a common estrogen-dependent gynecologic disorder, AM has been investigated using 16S rRNA sequencing of the gut microbiota in patients and healthy controls. Findings revealed significantly reduced α-diversity in the gut microbiota of patients with AM, and β-diversity analyzes indicate distinct microbial community structures relative to healthy controls. Animal studies corroborate these observations: AM model mice exhibit gut compositional shifts characterized by a significantly higher Firmicutes-to-Bacteroidetes ratio and increased relative abundance of lactobacilli compared with controls (Chen et al., 2023; Valdés-Bango et al., 2024). However, research on the relationship between AM and the GM faces several challenges, primarily due to the limited availability of large, multicenter clinical datasets, which hinders a comprehensive understanding of this association. Additionally, insufficient characterization of the interplay between microbial composition and functional activity presents a further challenge. Notably, prior studies have not systematically evaluated causal links between GM and AM.

This study employs Mendelian randomization to infer causal links between the gut microbiota and AM and to investigate whether immune cell phenotypes mediate this association. Mendelian randomization, as a method for inferring causality second only to randomized controlled trials, primarily leverages genetic variants as instrumental variables to infer causal associations between exposure factors and outcomes. Since these genetic variants are determined at conception, this method offers unique advantages in controlling for confounding factors and avoiding reverse causality (He et al., 2025). In recent years, with the continuous accumulation of genome-wide association study (GWAS) data, Mendelian randomization has been increasingly applied in the study of complex disease mechanisms, offering a new perspective for exploring the causal relationship between the gut microbiota and adenomyosis. Although preliminary progress has been made in identifying associations between gut microbiota and adenomyosis, the potential mediating role of immune cells in this relationship warrants further investigation (Tang et al., 2024). Furthermore, the ongoing updates to GWAS data underscore the necessity for further analysis. Furthermore, we systematically explore the potential impact of specific gut microbial taxa on the development of AM.

We employed MR to explore causal relationships between the gut microbiota and its metabolic pathways with AM, and to assess the mediating role of immune cells in this association. Utilized metagenomic sequencing, we compared gut microbiota profiles in women with AM versus healthy controls and conducted validation analyzes to corroborate identified associations. Our study provides a robust evidence base for developing novel preventative and therapeutic interventions for AM.

## Methods

### Two-sample Mendelian randomization and mediational Mendelian randomization

Genetic data on the gut microbiome were obtained from a comprehensive genome-wide association study (GWAS) conducted by the Netherlands Microbiome Project consortium (Lopera-Maya et al., 2022), involving 7,738 individuals of European ancestry. After rigorous quality control and direct classification, the study assessed associations between 558,468 Single nucleotide polymorphisms(SNPs) and gut microbiome features. The dataset included a hierarchical taxonomy comprising 5 phyla, 10 classes, 13 orders, 26 families, 48 genera, and 105 species, totaling 207 annotated microbial taxa. Additionally, functional genomics analyzes identified 205 pathways associated with microbial metabolic processes, disclosed gut microbiota biology at the gene-function level.

Genetic variants associated with AM were sourced from the FinnGen project, which included 5,130 cases and 119,468 controls, encompassing a total of 21,298,398 SNPs (Kurki et al., 2023). AM cases were identified using International Classification of Diseases (ICD) codes from versions 8, 9, and 10, with primary ascertainment based on ICD-10.

Data from a GWAS of 3,757 Sardinian individuals yielded 731 immune features: 118 absolute cell counts (ACs), 389 surface antigen expression levels (e.g., median fluorescence intensity, MFI), 32 morphological parameters (MPs), and 192 relative cell counts (RCs). MFI, ACs, and RCs provide insights into the developmental stages of multiple immune cell types, including B cells, conventional dendritic cells (cDCs), T cells, monocytes, myeloid cells, TBNK cells (T cells, B cells, and natural killer cells), and regulatory T cells (Tregs). Notably, MPs capture morphological characteristics linked to cDCs and TBNK cells (Orrù et al., 2020).

We concentrated solely on European data to enhance statistical power and to reduce the potential of population stratification bias.

### Screening of instrumental variables

The selection of instrumental variables follows these criteria: Due to the limited number of gut microbiota-associated SNPs identified at the genome-wide significance level (*p *< 5 × 10^-8^), a more lenient threshold (*p* < 1 × 10^-5^) was adopted for SNP selection. This is followed by linkage disequilibrium clustering analysis (r² = 0.001, kb = 10,000). Finally, weak instrumental variables with F-statistics below 10 are excluded to ensure the strength of genetic variation (Burgess et al., 2017). The calculations for R² and F-statistics are performed using the following formulas:


$$R^{2}=\frac{2\times \beta^{2}\times EAF\times (1-EAF)}{2\times EAF\times (1-EAF)\times \beta^{2}+2\times EAF\times (1-EAF)\times N\times SE^{2}}$$

$$F=\frac{R^{2}\times (N-2)}{\left(1-R^{2}\right)}$$

### Statistical analysis and sensitivity analysis

A two-sample MR analysis was performed using GWAS data on gut microbiota and AM to investigate potential causal relationships in two directions: (1) gut microbiota as the exposure and AM as the outcome; and (2) AM as the exposure and gut microbiota as the outcome. This bidirectional design helps reduce the likelihood of reverse causation.

To assess causality, we used IVW as the primary estimator. IVW combines Wald estimates from individual SNPs with inverse-variance weighting and is consistent under the assumption that all instruments are valid and not affected by horizontal pleiotropy (Burgess et al., 2013). IVW synthesizes Wald odds-ratio estimates from SNPs using a fixed-effects meta-analysis, with weights equal to the inverse of the estimated variance. This approach yields efficient causal effect estimates when genetic instruments are valid. We applied a significance threshold of *p*< 0.05 to identify statistically significant associations. We assessed horizontal pleiotropy—where SNPs may affect the outcome through pathways unrelated to the exposure—using the MR-Egger intercept test. We quantified heterogeneity among SNPs using the Cochran Q test to evaluate the consistency of SNP-level estimates (Verbanck et al., 2018). In cases MR analysis results were statistically significant, the gut microbiota were classified as risk or protective factors by the Odds ratios(OR).

### Mediation analysis

Mediation analysis aims to investigate whether a specific exposure influences an outcome through an intermediary variable, thereby providing evidence for uncovering the underlying mechanisms linking exposure factors to outcomes. This approach facilitates exploration of the potential pathways through which exposure affects outcomes (Carter et al., 2021). Initially, univariable Mendelian randomization was employed to quantify the impact of the exposure on the mediator, yielding the regression coefficient β1. Subsequently, multivariable MR analysis was conducted to determine the effect of each mediator on the outcome, independent of the exposure and other mediators, producing the coefficient β2. The indirect effect, representing the mediated pathway, was then calculated as the product of these two coefficients (β1 × β2) (Carter et al., 2021). To elucidate the mediating role of immune cells, we employed this model to estimate the proportion of the overall effect of gut microbiota on AM that is mediated by immune cells.

All analyzes were conducted using the TwoSampleMR and MR-PRESSO packages within the R software environment (version 4.3.2).

### Study population

We conducted a comparative study involving twenty-two patients with isolated AM or adenomyoma who received care in the inpatient department or physical examination center of the Third Affiliated Hospital of Zunyi Medical University between January 2024 and December 2024; these patients constituted the AM group. Additionally, twenty-three volunteers served as the healthy control group.

### Inclusion criteria

The AM group was classified according to the 2023 criteria of the Society of Obstetricians and Gynaecologists of Canada (SOGC) (Dason et al., 2023). Preoperative diagnoses were made using ultrasound or MRI and were confirmed postoperatively by pathological examination. The Control group comprised healthy women matched by age. All participants had resided in Zunyi City, Guizhou Province, China, or nearby areas for at least six months prior to enrollment.

### Exclusion criteria

1. Gastrointestinal disorders, including gastrointestinal hemorrhage, inflammatory bowel disease, or bacterial dysentery;
2. Coexisting conditions, including estrogen-related gynecologic disorders (e.g., uterine fibroids, endometriosis), malignancies, endocrine disorders (e.g., hyperthyroidism or Cushing syndrome), or other autoimmune diseases; severe dysfunction of vital organs, including the heart, liver, brain, or kidneys;
3. Fever or active infectious diseases (e.g., pulmonary infection, urinary tract infection);
4. Gastrointestinal symptoms within four weeks before enrollment, such as nausea, vomiting, diarrhea, or abdominal pain;
5. Use of antibiotics, probiotics, or related agents within four weeks before enrollment.

### Fecal sample collection, storage, and metagenomic sequencing

After morning defecation into a sanitized commode, trained personnel will collect a fresh stool sample (approximately 3–4 g) from the internal portion using a sterile, clinical-grade collection device. The sample will be promptly aliquoted into pre-labeled cryogenic tubes and stored at −80 °C in an ultra-low-temperature freezer.

Prepare five 96-well deep-well plates and pre-dispense reagents as follows: In the first well, add 625 μL of the magnetic-bead binding mix consisting of 600 μL Binding Buffer, 20 μL Proteinase K, and 5 μL RNase A. In the next three wells, dispense 700 μL each of Wash Buffers 1, 2, and 3, respectively. In the last well, add 100 μL Elution Buffer. Accurately weigh 100–200 mg of sample and transfer it into a tube preloaded with bead-beating media. Add 1 mL of an ATL buffer/PVP-10 mixture to the tube. Homogenize thoroughly using a high-speed bead beater or homogenizer. Incubate at 65 °C for 20 min. Centrifuge the lysate at 14,000 × g for 5 min. Transfer the supernatant to a new tube. Add 0.6 mL PCI (phenol:chloroform:isoamyl alcohol) solution. Vortex for 15 s. Centrifuge at 18,213 × g for 10 min. Transfer the supernatant to the first well of the preloaded magnetic-bead binding plate. Start the KingFisher instrument and run the designated program, ensuring plates are loaded in the specified order for the workflow. Upon program completion, transfer the eluate from the final well into a 1.5 mL microcentrifuge tube for storage at −20 °C (short-term) or −80 °C (long-term).

After a senior outpatient physician confirms a diagnosis of adenomyosis via ultrasound during the outpatient visit and issues a corresponding referral, stool samples are collected at the inpatient ward using the method described in the text, appropriately labeled, and stored. Once postoperative pathology results become available, stool samples are selected for testing exclusively from cases with a final histopathological diagnosis of isolated adenomyosis or uterine leiomyoma (fibroids).

Library preparation employed the BGI Optimal DNA Library Prep Kit (BGI, Shenzhen, China). Approximately 200 ng of genomic DNA was fragmented and purified by magnetic bead selection. End repair, 3′ A-tailing, adapter ligation, and PCR amplification were performed on the reaction mixture. Library quality was assessed by fluorescent dye-based quantification. Libraries that met quality criteria were denatured to generate single-stranded DNA molecules. A circularization reaction was used to produce single-stranded circular products, with residual linear DNA removed. Circular DNA molecules underwent phi29-mediated rolling circle replication to form DNA nanoballs (DNBs) containing multiple copies. DNBs were loaded into mesh-wells on high-density DNA nanochips. Sequencing was performed on the DNBSEQ-G400T10 platform using combined probe-anchored polymerization sequencing (cPAS) with 150-bp paired-end reads.

Species annotation was performed using Kraken 2 with default parameters. Species-level abundances in metagenomic samples were then estimated with Bracken based on Kraken classifications using a Bayesian framework. The Unified Human Gastrointestinal Genome (UHGG) database served as the reference (Almeida et al., 2021). Alpha diversity metrics were calculated, including the Chao1 index, Shannon index, and Simpson index. Furthermore, Bray-Curtis distance (Bray and Curtis, 1957) and Jensen-Shannon divergence (JSD) were utilized to assess dissimilarities between samples or groups (Majtey et al., 2005), representing Beta diversity (Whittaker, 1960), to ascertain the presence of significant microbial community distinctions between samples or groups.

LEfSe (LDA Effect Size) was applied to identify and interpret biomarkers (including genes, pathways, and taxonomic units) from high-dimensional data, allowing comparisons across two or more groups. This method prioritizes both statistical significance and biological relevance to detect biomarkers with significant intergroup differences. The analysis followed three key steps: (1) the Kruskal-Wallis rank-sum test was first used to identify features with significantly differential abundance across groups; (2) pairwise comparisons of these differential features were then performed using the paired Wilcoxon rank-sum test; (3) finally, Linear Discriminant Analysis (LDA) was conducted to estimate the effect size of differential taxa on group separation, with results reported as LDA scores. Furthermore, the ANCOM-BC approach was utilized to validate the statistically differential taxa that were simultaneously identified by both LEfSe and Mendelian randomization (MR) analyzes.

## Result

### Genetic causality between gut microbiota and AM

**Figure 1:**
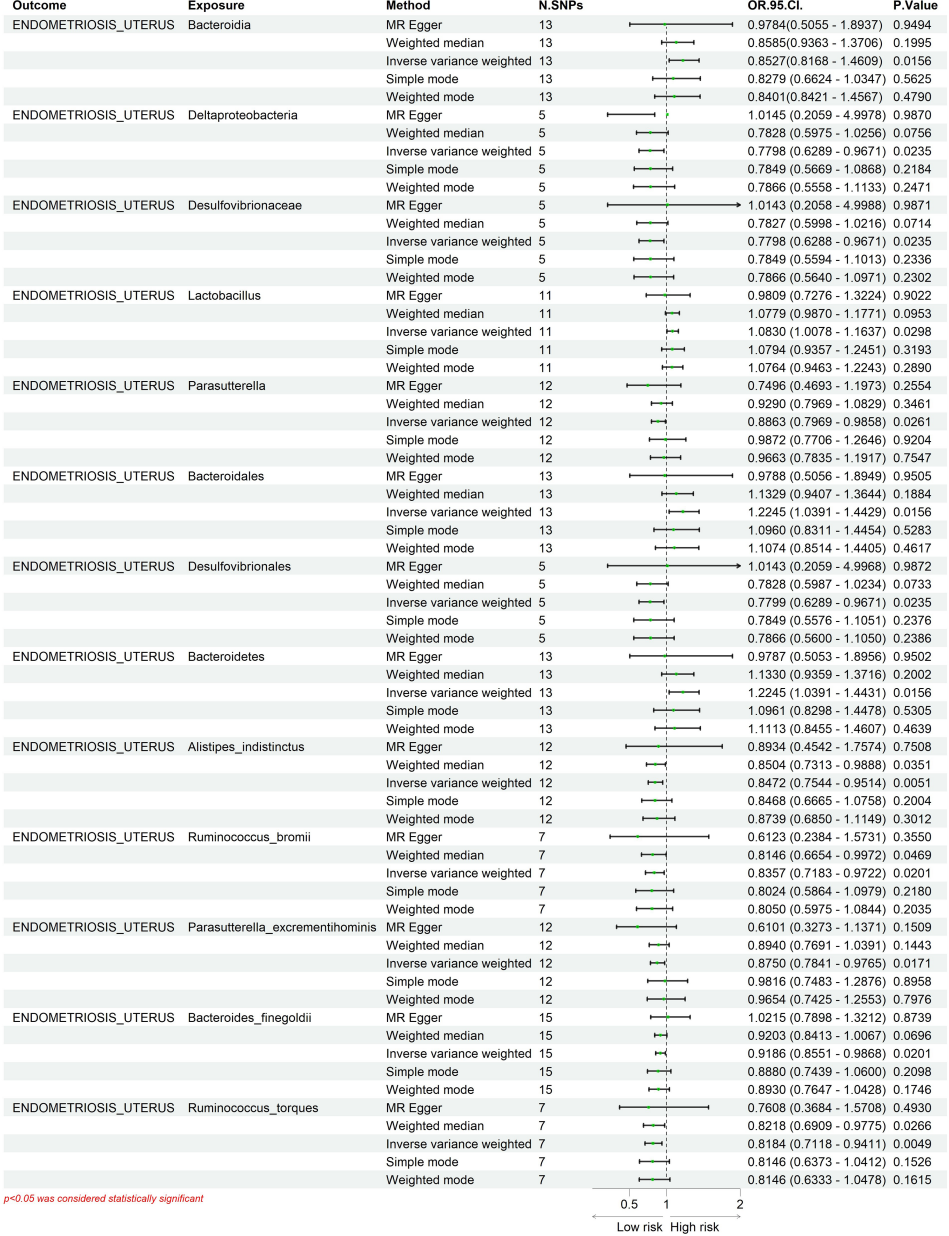
Mendelian randomization results of causal effects between microbial taxa and adenomyosis. ENDOMETRIOSIS_UTERUS, adenomyosis; IVW, inverse variance weighted; Nsnp, number of single nucleotide polymorphism; OR, odds ratio.

In a two-sample Mendelian randomization analysis, we investigated the relationship between the gut microbiota and AM. Using IVW, we identified twelve gut microbial taxa significantly associated with AM. The taxa with decreased risk were: *Alistipes indistinctus* (OR = 0.8472, 95% CI = 0.7544-0.9514, *p* = 0.005,*p*FDR>0.05), *Ruminococcus torques* (OR = 0.8184, 95% CI = 0.7118-0.9411, *p* = 0.005,*p*FDR>0.05), *Deltaproteobacteria* (OR = 0.7798, 95% CI = 0.6289-0.9671, *p* = 0.024,*p*FDR>0.05), *Desulfovibrionaceae* (OR = 0.7798, 95% CI = 0.6288-0.9671, *p* = 0.024,*p*FDR>0.05), *Desulfovibrionales* (OR = 0.7799, 95% CI = 0.6289-0.9671, *p* = 0.024,*p*FDR>0.05), *Parasutterella excrementihominis* (OR = 0.8750, 95% CI = 0.7841-0.9765, *p* = 0.017,*p*FDR>0.05), *Ruminococcus bromii* (OR = 0.8357, 95% CI = 0.7183-0.9722, *p* = 0.020,*p*FDR>0.05), *Bacteroides finegoldii* (OR = 0.9186, 95% CI = 0.8551-0.9868, *p* = 0.020,*p*FDR>0.05), and *Parasutterella* (OR = 0.8863, 95% CI = 0.7969-0.9858, *p* = 0.026).Additionally, taxa associated with increased risk included *Bacteroidales* (OR = 1.2244, 95% CI = 1.0391-1.4429, *p* = 0.016,*p*FDR>0.05), *Lactobacillus* (OR = 1.0830, 95% CI = 1.0078-1.1637, *p* = 0.030,*p*FDR>0.05), and *Bacteroidetes* (OR = 1.2245, 95% CI = 1.0391-1.4431, *p* = 0.016,*p*FDR>0.05). We have verified that no SNPs located within the LCT (Chr2) or ABO (Chr9) regions are included (**Figure 1**; **Supplementary Table 1**). We have verified that no SNPs located within the LCT (Chr2) or ABO (Chr9) regions are included.

To assess the robustness of the results, we performed multiple sensitivity analyzes. The MR-Egger intercept test and Cochran’s Q indicated no evidence of pleiotropy or heterogeneity. However, reverse MR suggested inverse causal effects of *Bacteroidales* and *Bacteroidetes* on AM, indicating that these taxa are unsuitable for inclusion (**Supplementary Tables 2**, **S5**).

### Genetic causality between gut microbiota metabolic pathways and AM

**Figure 2:**
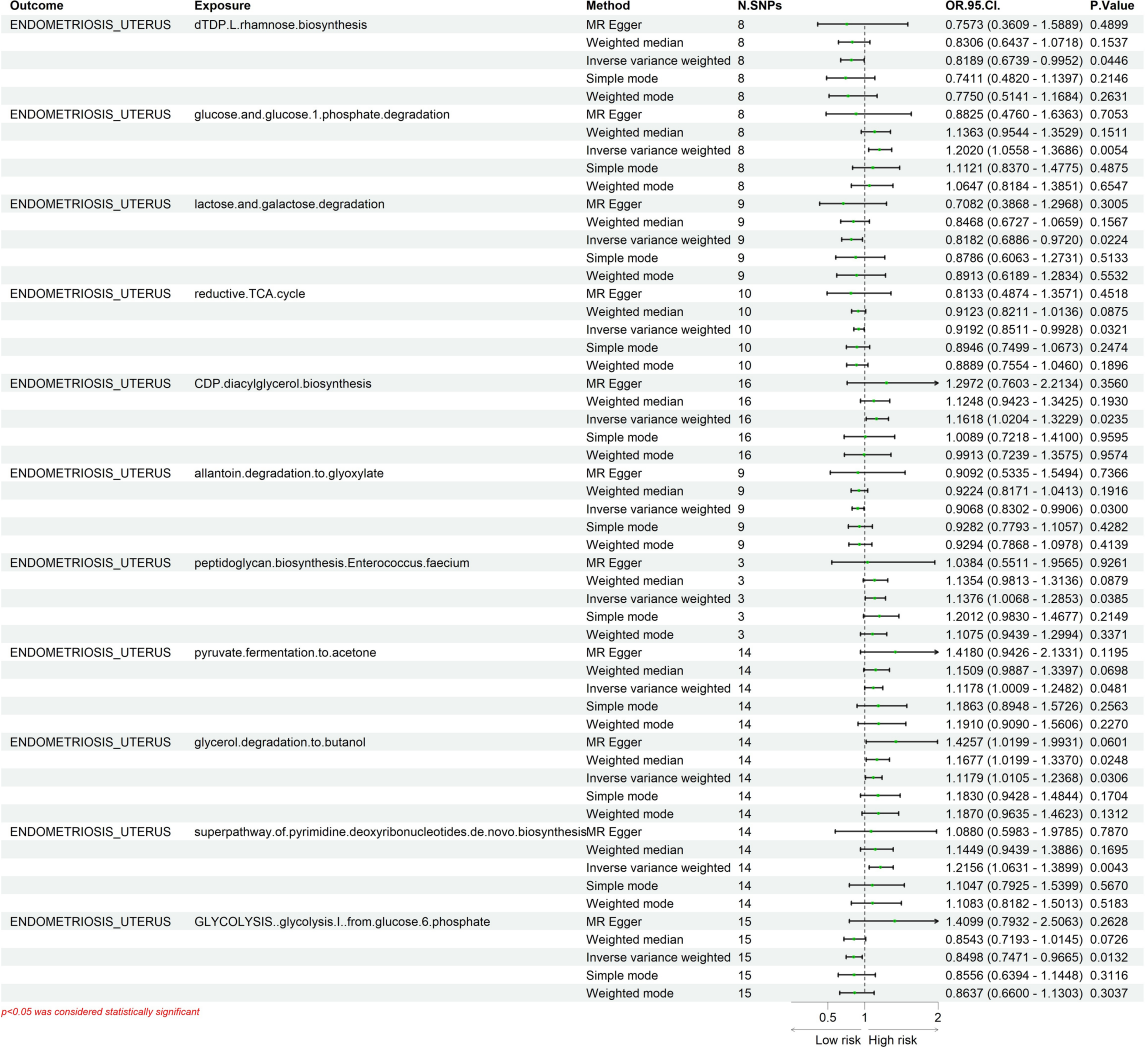
Mendelian randomization results of causal effects between Microbial metabolic pathways and adenomyosis. ENDOMETRIOSIS_UTERUS, adenomyosis; IVW, inverse variance weighted; Nsnp, number of single nucleotide polymorphism; OR, odds ratio.

Subsequently, we conducted a two-sample MR analysis using gut microbiota metabolic pathways as the exposure and AM as the outcome. Using IVW, we identified eleven significant associations between gut metabolic pathways and AM. Five pathways were associated with decreased risk:dTDP-L-rhamnose biosynthesis (OR = 0.8189, 95% CI = 0.6739-0.9952, *p* = 0.0446,*p*FDR>0.05), lactose and galactose degradation (OR = 0.8182, 95% CI = 0.6886-0.9720, *p* = 0.0224,*p*FDR>0.05), reductive TCA cycle (OR = 0.9192, 95% CI = 0.8511-0.9928, *p* = 0.0321,*p*FDR>0.05), allantoin degradation to glyoxylate (OR = 0.9068, 95% CI = 0.8302-0.9906, *p* = 0.0300,*p*FDR>0.05), and glycolysis I from glucose-6-phosphate (OR = 0.8498, 95% CI = 0.7471-0.9665, *p* = 0.0132,*p*FDR>0.05). Six pathways were associated with increased risk: degradation of glucose and glucose-1-phosphate (OR = 1.2020, 95% CI = 1.0558-1.3686, *p* = 0.0054,*p*FDR>0.05), CDP-diacylglycerol biosynthesis (OR = 1.1618, 95% CI 1.0204-1.3229, *p* = 0.0235,*p*FDR>0.05), peptidoglycan biosynthesis in Enterococcus faecium (OR = 1.1376, 95% CI = 1.0068-1.2853, *p* = 0.0385,*p*FDR>0.05), pyruvate fermentation to acetone (OR = 1.1178, 95% CI = 1.0009-1.2482, *p* = 0.0481,*p*FDR>0.05), glycerol degradation to butanol (OR = 1.1179, 95% CI = 1.0105-1.2368, *p* = 0.0306,*p*FDR>0.05), and *de novo* pyrimidine deoxyribonucleotide biosynthesis (OR = 1.2156, 95% CI = 1.0631-1.3899, *p* = 0.0043,*p*FDR>0.05). We have verified that no SNPs located within the LCT (Chr2) or ABO (Chr9) regions are included (**Figure 2**; **Supplementary Table 3**). We have verified that no SNPs located within the LCT (Chr2) or ABO (Chr9) regions are included.

Quality control showed no evidence of pleiotropy (*p*>0.05) or heterogeneity (*p*>0.05) by MR-Egger regression and Cochran’s Q, supporting the robustness of the findings (**Supplementary Table 4**).

Furthermore, reverse MR suggested an inverse causal effect of the CDP-diacylglycerol biosynthesis pathway on AM, so this pathway was excluded as unsuitable for inclusion (**Supplementary Table 5**).

### Effects of immune cell characteristics on AM

Using IVW, we identified protective effects of five immune cell types against AM. In contrast, nineteen genetically predicted immune cell traits were associated with increased risk of AM. *P*-values from Cochran’s Q for both IVW and MR-Egger were above 0.05, indicating no significant heterogeneity. Additionally, the MR-Egger intercept and MR-PRESSO were non-significant, suggesting no evidence of pleiotropy. Leave-one-out analysis showed that excluding individual SNPs did not materially affect the causal estimates. (**Supplementary Tables 6**–**S8**) We have verified that no SNPs located within the LCT (Chr2) or ABO (Chr9) regions are included. We have verified that no SNPs located within the LCT (Chr2) or ABO (Chr9) regions are included.

### The effects of gut microbiota and their metabolic pathways on immune cells

Using IVW we identified protective effects of eleven gut microbiota species and their associated pathways on immune cell function. In contrast, thirteen genetically predicted gut microbiota species and their pathways were associated with increased risk of immune cell traits. P-values from Cochran’s Q for both IVW and MR-Egger were >0.05, indicating no significant heterogeneity. Furthermore, the MR-Egger intercept and MR-PRESSO were non-significant, indicating no evidence of pleiotropy. Leave-one-out analysis showed that excluding individual SNPs did not materially affect the causal estimates. We have verified that no SNPs located within the LCT (Chr2) or ABO (Chr9) regions are included (**Supplementary Tables 9**–**S11**) We have verified that no SNPs located within the LCT (Chr2) or ABO (Chr9) regions are included.

### Mediation analysis

In the final analysis, we conducted mediation analyzes to clarify causal pathways linking gut microbiota and their metabolic pathways to AM via immune cell phenotypes. We found that the effect of *Ruminococcus bromii* on AM was significantly mediated by CD24 expression on CD24+CD27+ cells (*p*< 0.05). This mediation in the protective pathway from *Ruminococcus bromii* to AM accounted for 32.91% of the total effect (**Supplementary Table 12**).

### Descriptive characteristics of the study participants

Our study population of 45 women consisted of a total of 22 women with adenomyosis and 23 control women. The descriptive characteristics of study participants are summarized in Table. No significant differences were observed between the two groups regarding age (years), parity, weight (kg), height (cm), BMI, or history of gynecological surgery (*p* > 0.05). In contrast, the AM group exhibited significantly higher rates of pregnancy, dysmenorrhea, and menstrual irregularities than the control group (*p*< 0.05) (**Supplementary Table 13**).

### Diversity analysis in metagenomic sequencing

**Figure 3:**
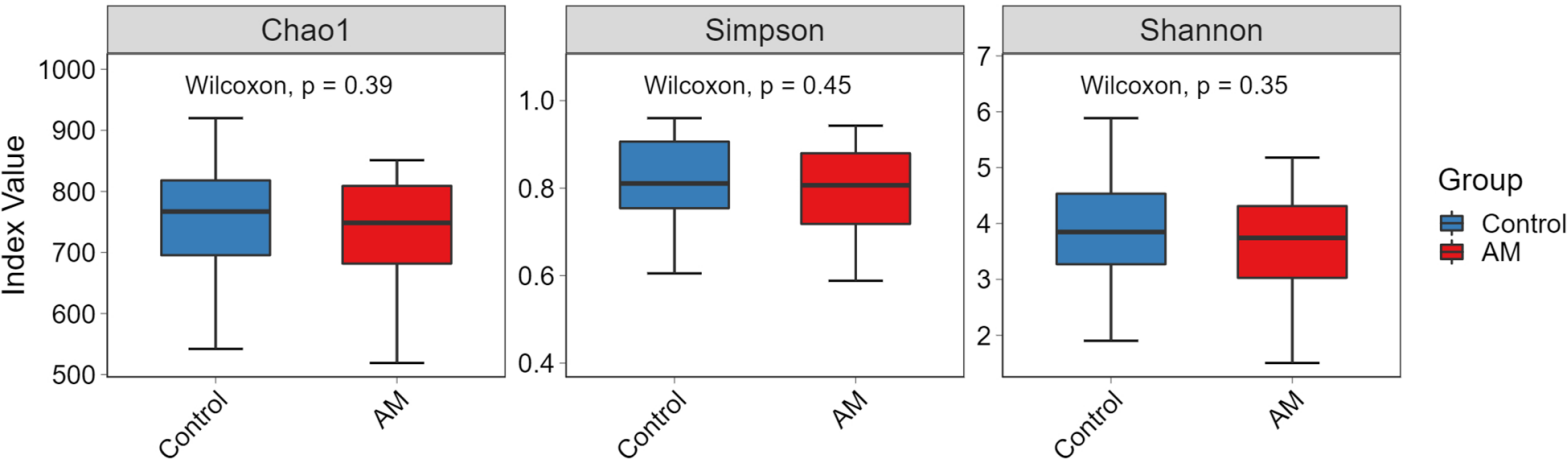
Boxplot of species alpha diversity. Each box plot represents a diversity index. The x-axis and distinct boxes denote different groups, while the y-axis shows index values. The upper left corner of each box plot indicates the hypothesis testing method and result, where *p* < 0.05 signifies significant differences in Alpha indices between groups. When the number of groups is 3 or 4, a horizontal line above the bars connects two groups, displaying either the statistical test symbol or p-value above the line: **** indicates *p* < 0.0001, *** indicates *p* < 0.001, ** indicates *p* < 0.01, * indicates *p* < 0.05, ns indicates *p* > 0.05, and NS indicates *p* = 1.

**Figure 4:**
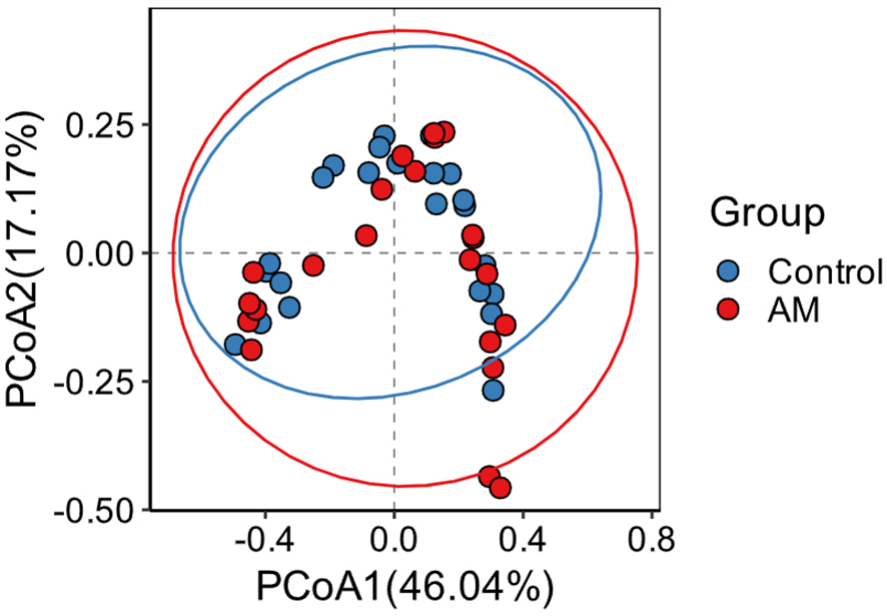
Each point represents a sample; the color of the point indicates the group to which the sample belongs; the x- and y-coordinates represent the two dimensions—PCoA1 and PCoA2—that provide the highest explanatory power after dimensionality reduction; the numbers in parentheses on the axis labels indicate the explanatory power of each dimension after dimensionality reduction; and the ellipses represent 95% confidence intervals.

**Figure 5:**
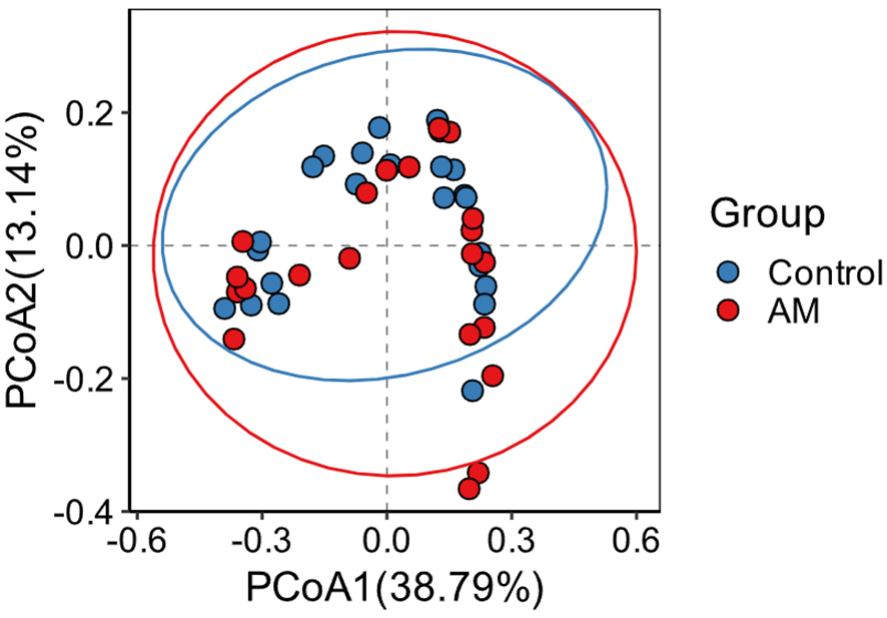
Each point represents a sample; the color of the point indicates the group to which the sample belongs; the x- and y-coordinates represent the two dimensions—PCoA1 and PCoA2—that provide the highest explanatory power after dimensionality reduction; the numbers in parentheses on the axis labels indicate the explanatory power of each dimension after dimensionality reduction; and the ellipses represent 95% confidence intervals.

At the genus level, α-diversity in gut microbiota showed no significant difference between patients with AM and healthy females *p*. Similarly, at the species level, β-diversity did not differ significantly between the two groups (**Figures 3**–**5**).

### Analysis of species differences in metagenomic sequencing

**Figure 6:**
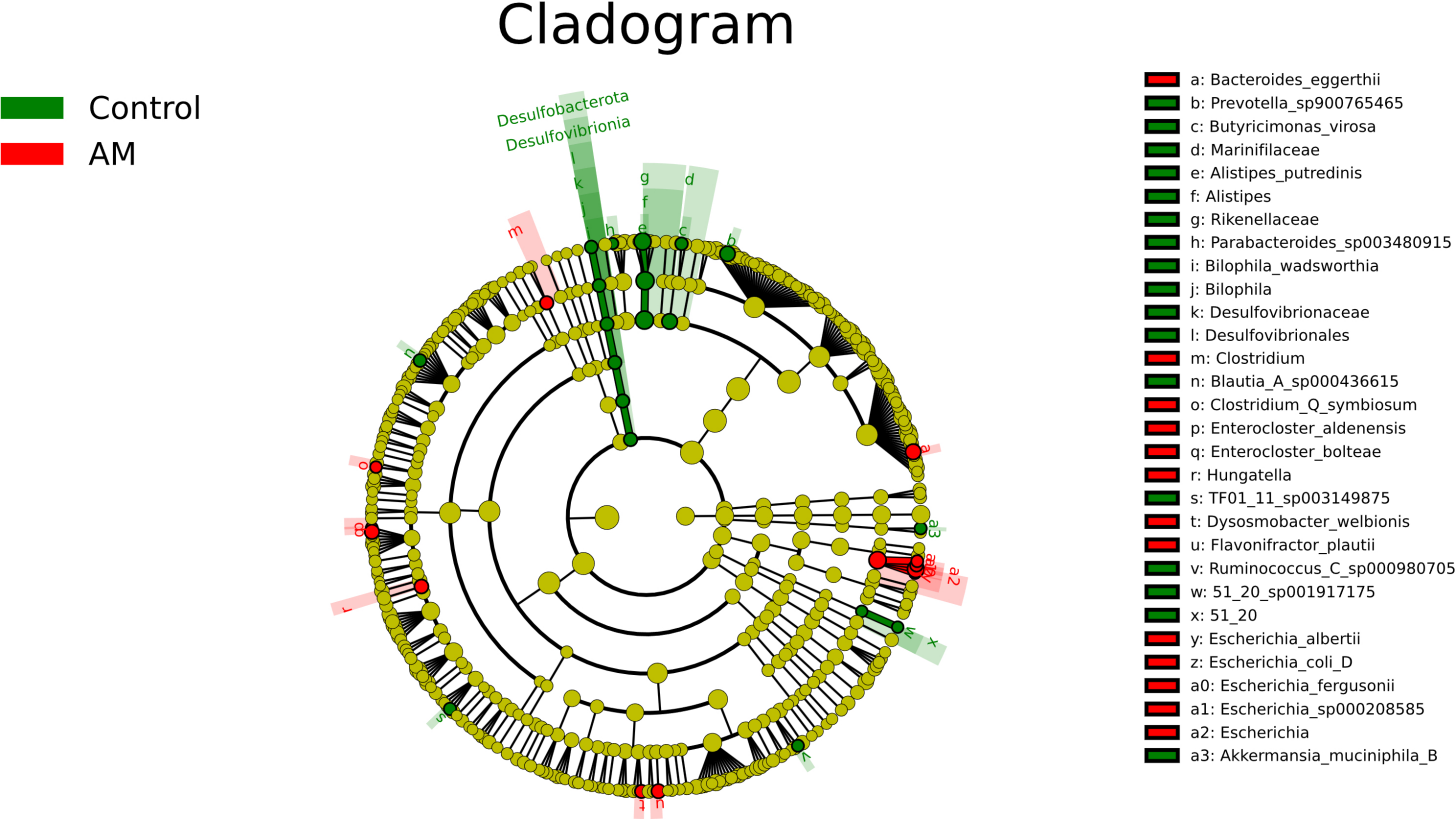
An evolutionary tree diagram is a circular diagram composed of multiple rings, with the inner ring representing higher taxonomic levels and the outer ring representing lower taxonomic levels. Each point represents a specific species classification, with point size indicating relative abundance. Points for undifferentiated species classifications appear yellow, while significantly differentiated classifications are colored by group. If a group lacks a significantly differentiated species with the highest abundance, that group is not displayed on the diagram. Points colored by group indicate that the corresponding microbial classification plays a significant role within that group. The color of the sector represents the higher-level species classification. Significantly different classifications not labeled in the figure are marked with letters within the diagram, with corresponding names displayed in the upper-right corner of the image.

To visualize group differences in gut microbiota abundance, LEfSe was applied across taxonomic ranks from phylum to species. **Figure 6** presents a cladogram that uses color coding to display differential taxa. Red nodes indicate taxa enriched in the AM group, predominantly within *Escherichia*, including *Escherichia coli* and *Escherichia albertii*. In contrast, taxa such as *Bacteroides eggerthii*, *Clostridium*, *Enterocloster bolteae*, *Hungatella*, and *Flavonifractor plautii* show dispersed patterns. Green nodes mark taxa characteristic of the Control group, with notable enrichment along *Desulfobacterota* and taxa such as *Rikenellaceae*, *Alistipes*, and *Marinifilaceae*. Yellow nodes indicate taxa without statistically significant differences (**Figure 6**).

**Figure 7:**
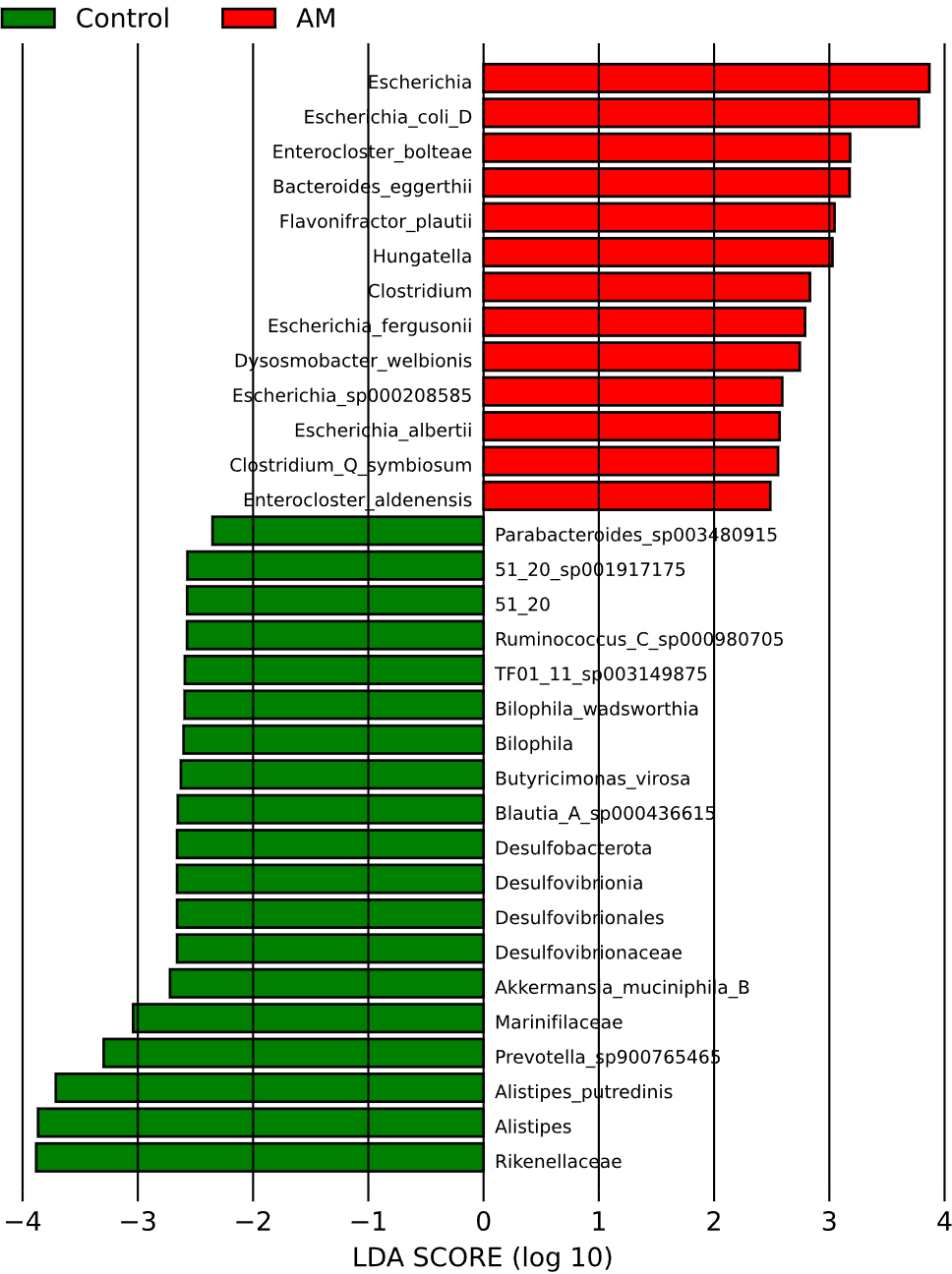
The vertical axis represents distinct classification units, while the horizontal axis displays LDA values. Higher LDA values indicate greater contribution to intergroup differences. Column colors denote groupings, and the image only shows classifications with LDA values exceeding a specified threshold (typically 2). Positive or negative values on the horizontal axis merely indicate direction.

To provide a comprehensive analysis, group differences were quantified by LDA scores. An LDA score threshold of above two was used to identify discriminatory biomarkers. A bar plot of LDA scores was generated to visualize each species’ contribution to between-group differences. The figure displays species with LDA scores >2; bar length reflects relative discriminatory effect (**Figure 7**). The comprehensive results of differential abundance analysis (p-values) are presented in **Supplementary Table 14**.

In the AM group (red bars), taxa with increased abundance and greater discriminatory impact, ranked from highest to lowest, include *Enterocloster aldenensis, Escherichia albertii, Clostridium Q symbiosum, Escherichia sp00208585, Escherichia fergusonii, Dysosmobacter welbionis, Clostridium, Hungatella, Flavonifractor plautii, Enterocloster bolteae, Bacteroides eggerthii, Escherichia coli D*, and *Escherichia*. Additionally, the AM group showed increased abundance in the red bar chart. Taxa contributing most to the differences, ranked by impact from highest to lowest, were *Rikenellaceae, Alistipes, Alistipes putredinis, Prevotella* sp. *900765465, Marinifilaceae, Akkermansia muciniphila B, Blautia A* sp. *000436615, Butyricimonas virosa, 51_20, 51_20_sp001917175, Desulfovibrionaceae, Desulfovibrionales, Desulfovibrionia, Desulfobacterota, TF01_11_sp003149875, Bilophila, Ruminococcus C sp000980705, Bilophila wadsworthia*, and *Parabacteroides sp003480915*. All observed differences were statistically significant (*p*< 0.05).

After adjusting for confounding factors including age and BMI, ANCOM-BC differential abundance analysis was performed on microbial taxa across all taxonomic levels (kingdom, phylum, class, order, family, genus, and species). The results (**Supplementary Table S15**–**S20**) showed that *Desulfovibrionaceae* and *Desulfovibrionales* remained statistically significantly different between groups (*p*< 0.05).

## Discussion

This study represents, to our knowledge, the first application of Mendelian randomization to systematically evaluate causal relationships among gut microbiota, microbial metabolic pathways, immune-cell phenotypes, and AM. We identified thirteen microbial taxa and eleven metabolic pathways with nominally significant causal associations with AM. After exclusion of taxa and pathways exhibiting evidence of reverse causation in bidirectional MR, ten taxa and ten pathways were retained for downstream analysis. In the forward MR framework (gut microbiota → immune cells), eleven exposure–outcome pairs demonstrated protective effects and thirteen were associated with increased risk. In the reverse direction (immune cells → AM), five immune traits showed protective effects and nineteen were associated with increased risk. Mediation analysis revealed that CD24 expression on CD24^+^CD27^+^ B cells mediated the protective pathway from *Ruminococcus bromii* to AM, accounting for 32.91% of the total effect. In a complementary clinical cohort of Chinese women, shotgun metagenomic sequencing replicated key taxonomic signals — notably *Desulfovibrionaceae* and *Desulfovibrionales* — that were consistent with the MR findings, whereas pathway-level associations were not reproduced, potentially reflecting ancestry differences or limited sample size.

Our comprehensive approach underscores the pivotal role of the gut-immune axis in the development of AM, and identifies microbial and immune targets for prevention and therapy. In our study, *Alistipes indistinctus*, a prominent Gram-negative member of *Bacteroidetes*, enhances intestinal barrier integrity, suppresses TLR/NF-κB signaling, and promotes beneficial microbial populations (Gacesa et al., 2022; Xu et al., 2024). *Ruminococcus torques* produces 2-hydroxy-4-methylpentanoate (HMP), which inhibits the gut HIF-2α-ceramide pathway, thereby reducing inflammation and fibrosis, enhancing carbohydrate-metabolizing enzyme activity, and increasing short-chain fatty acid production (Wang et al., 2023a; Zhou et al., 2024). *Ruminococcus bromii* excels in starch degradation, stimulates butyrate production through symbiotic interactions, regulates inflammation by inhibiting histone deacetylases, activates GPR109A/GPR41/GPR43, strengthens the epithelial barrier, increases antimicrobial peptide secretion, modulates the microbial community, and exerts antioxidative effects (Ze et al., 2015; Couto et al., 2020; Lordan et al., 2020). *Parasutterella* plays a role in butyrate synthesis and, via GPR/HDAC3 and LPS/TLR4/NF-κB signaling pathways, rectifies Th17/Treg imbalance and reinstates intestinal integrity (Wang et al., 2024). Several animal studies involving the subspecies *Parasutterella_excrementihominis* have shown a positive association with pyruvate, accompanied by reduced inflammation in the uterus and placenta. Furthermore, through tryptophan-metabolizing pathways, it exerts anti-inflammatory and anxiolytic effects (Galley et al., 2024; Jia et al., 2024).

Contrary to its commonly probiotic function, *Lactobacillus* has been associated with increased risk. High levels of Lactobacillus may disrupt estrogen homeostasis by altering β-glucuronidase and β-glucosidase in the enterohepatic circulation (Sui et al., 2021). This promotes estrogen reabsorption and may increase the risk of AM. Animal studies have shown higher gut Lactobacillus levels in AM models than in controls (Rastogi and Singh, 2022; Chen et al., 2023; Wang et al., 2023b; Liu et al., 2024; Zancan et al., 2024). Our findings also indicate a role for microbial metabolic pathways—particularly lactose and galactose degradation—in shaping the uterine microenvironment via the gut-uterus axis. Mechanistically, dietary lactose is hydrolyzed by β-galactosidase (e.g., from Lactobacillus intestinalis) into galactose. Subsequent galactose absorption may activate C/EBPβ in endometrial epithelial cells, upregulating S100A8 and conferring anti-inflammatory effects within the uterine cavity (Cai et al., 2025). This may mitigate chronic inflammation at the endometrial-myometrial junction and preserve epithelial barrier integrity, thereby reducing the onset of AM.

Moreover, butyrate derived from *Ruminococcus bromii* may reduce the risk of AM by promoting Treg differentiation and reinforcing immunosuppression (Zhang et al., 2021). Our analysis suggests that *Ruminococcus bromii* may mitigate AM risk by suppressing CD24 on CD24+ CD27+ cells, thereby enhancing Treg activity and increasing IL-10 secretion (Shi et al., 2022). This finding points to a potential therapeutic avenue for AM. Despite the European ancestry of the Mendelian randomization datasets and the Chinese ethnics of our metagenomic cohort, we observed consistent presence of specific taxa (*Desulfovibrionaceae/Desulfovibrionales*) across ancestries. Prior studies have implicated these taxa in pathogenic processes, including hydrogen sulfide production, cholesterol uptake, and gallstone formation (Luo et al., 2023). However, other studies—such as the Guangdong gut-microbiome project—have reported inverse associations between the abundance of these taxa and Body Mass Index waist circumference, and uric acid, alongside positive associations with microbial diversity and the prevalence of beneficial taxa. These patterns are consistent with a potential protective role mediated by SCFA production and the maintenance of microbial equilibrium (Chen et al., 2021). These results suggest that microbial contributors to AM may beyond ethnic differences in need of validation in animal models and clinical studies.

The strengths of this study include being, to our knowledge, the first MR analysis to link gut microbiota, microbial metabolic pathways, and immune-cell phenotypes to AM. It provides a comprehensive assessment of 205 taxa, 207 pathways, and 731 immune traits, and integrates immune mediation analysis. Furthermore, microbial taxa were validated in an independent Chinese cohort.

This study has several limitations that warrant consideration. First, all fecal samples were collected after clinical diagnosis but prior to hormonal therapy or surgical intervention; consequently, the observed microbial differences may partly reflect the disease state itself or pain-induced physiological stress, and reverse causality cannot be entirely excluded. Second, the healthy control group comprised volunteers, introducing potential volunteer bias; unmeasured confounders such as dietary habits and health awareness were not fully accounted for. Third, the GWAS datasets underpinning the MR analyzes were predominantly derived from populations of European ancestry, which may limit the generalizability of our findings to other ethnic groups; moreover, the causal inferences derived from MR await mechanistic experimental validation. Fourth, the metagenomic cohort is relatively small, and replication in larger, independent cohorts is warranted.

Finally, the identified causal associations between gut microbiota and AM did not survive correction for multiple testing (*p*~FDR~ > 0.05). Nonetheless, given the exploratory nature of microbiome-wide MR studies and the inherently conservative behavior of the Benjamini–Hochberg procedure across 207 taxa, these nominal associations provide valuable suggestive evidence and biological plausibility for the gut–uterus axis, and merit further validation in larger cohorts.

## Conclusion

In summary, this study provides convergent evidence from Mendelian randomization and clinical metagenomic profiling that specific gut microbial taxa, their associated metabolic pathways, and immune-cell–mediated mechanisms are suggestively implicated in the pathogenesis of AM. Thirteen gut bacterial taxa and eleven microbial metabolic pathways demonstrated nominally significant causal associations with AM, and immune -cell phenotypes — notably CD24 expression on CD24^+^CD27^+^ B cells — may function as mediators along the gut microbiota–AM axis. These findings offer mechanistic insights into the pathogenesis of AM and identify candidate microbial and immune targets for future preventive and therapeutic strategies.

## Data Availability

The original contributions presented in the study are publicly available. This data can be found here: NCBI SRA, accession number PRJNA1473304.
